# To Be, or Not to Be … Pectoral Angina? The Pain Is the Same, but the Etiology Is Different—A Case Report

**DOI:** 10.3390/life14091066

**Published:** 2024-08-26

**Authors:** Ciprian Ilie Rosca, Daniel Florin Lighezan, Gabriel Veniamin Cozma, Horia Silviu Branea, Daniel Dumitru Nisulescu, Adrian Sebastian Zus, Stelian I. Morariu, Nilima Rajpal Kundnani

**Affiliations:** 1 Department of Internal Medicine I—Medical Semiotics I, Centre for Advanced Research in Cardiovascular Pathology and Haemostasis, “Victor Babeș” University of Medicine and Pharmacy, Eftimie Murgu Sq. No. 2, 300041 Timișoara, Romania; rosca.ciprian@umft.ro (C.I.R.); dlighezan@umft.ro (D.F.L.); 2 Department of Surgery I, Surgical Semiotics I and Thoracic Surgery, “Victor Babeș” University of Medicine and Pharmacy, Eftimie Murgu Sq. No. 2, 300041 Timișoara, Romania; gabriel.cozma@umft.ro; 3 Department of Internal Medicine I—Medical Semiotics II, “Victor Babeș” University of Medicine and Pharmacy, Eftimie Murgu Sq. No. 2, 300041 Timișoara, Romania; 4 General Medicine Faculty, “Victor Babeș” University of Medicine and Pharmacy, Eftimie Murgu Sq. No. 2, 300041 Timișoara, Romania; daniel.nisulescu@umft.ro; 5 General Medicine Faculty, ”Vasile Goldiș” West University Arad, 473223 Arad, Romania; 6 Department VI—Cardiology, “Victor Babeș” University of Medicine and Pharmacy, Eftimie Murgu Sq. No. 2, 300041 Timișoara, Romania; adrian.zus@umft.ro; 7 Discipline of Internal Medicine and Ambulatory Care, Prevention and Cardiovascular Recovery, Department of VI Cardiology, “Victor Babeș” University of Medicine and Pharmacy, 300041 Timișoara, Romania; knilima@umft.ro; 8 Research Centre of Timisoara Institute of Cardiovascular Diseases, “Victor Babeș” University of Medicine and Pharmacy, 3000041 Timișoara, Romania

**Keywords:** chest pain, angina pectoris, esophageal leiomyoma (EL), statins, aspirin, angio-CT

## Abstract

Background: Chest pain is one of the most common causes of emergency room visits and also accounts for numerous visits to the family physician’s office or Outpatient Clinics of cardiology or internal medicine. Case Report: Here we present a case of a 48-year-old female patient who presented to our hospital emergency unit but refused hospital admission. She presented in our Outpatient Clinic with a complaint of typical chest pain indicating it to be of coronary origin. A computed tomography (CT) coronary angiography for the evaluation of this chest pain was indicated. While ruling out the coronary origin of this chest pain, we were surprised to have incidentally identified the presence of an esophageal tumor mass that had intimate contact with carina of the trachea. After the diagnosis of esophageal leiomyoma was made and its surgical treatment was performed, the patient was asymptomatic. Approximately one year after the surgical intervention was performed, following the cessation of antiplatelet therapy and statin, the patient returned to our Outpatient Clinic complaining of chest pain again with the same characteristics as previously presented, being terrified by the possibility of the recurrence of the esophageal leiomyoma. Upon resuming investigations, it was proven through coronary angio-CT evaluation that the etiology of the chest pain was indeed coronary this time. However, the patient still refused hospital admission and the performance of percutaneous coronary angiography with the potential implantation of a coronary stent. Conclusions: Chest pain can be due to various underlying pathologies and should not be neglected. A thorough investigation and timely management are key to treating this possible fatal symptom. In our case, the patient presented twice with the complaint of typical chest pain indicating a possible coronary event, but at the first presentation, it was due to esophageal leiomyoma, while a year later, the patient had similar pain, which was indeed this time due to coronary blockage. Hence, it is of utmost importance to think of all possible scenarios and to investigate accordingly, leaving no stone unturned.

## 1. Introduction

Chest pain is one of the most commonly encountered symptoms of presentation addressed to the emergency room [1] being also responsible for frequent visits to the family doctor’s office [2] or Outpatient Clinics of cardiology or internal medicine.

Acute chest pain is defined as a painful or discomforting sensation in the chest, arising in a non-traumatic context, occurring in the last 24 h, localized in the anterior part between the base of the nose and the umbilicus and posteriorly between the occiput and the twelfth thoracic vertebra [3].

A high percentage of these patients with chest pain may have acute coronary syndrome (ACS) [4].

The exclusion of non-coronary causes of chest pain is the gold standard in approaching these patients and should be performed as soon as possible from the moment of presentation, followed by the evaluation of other possible causes in the order of their frequency. After excluding life-threatening causes, the diagnosis of non-cardiac chest pain (NCCP) is made, which is essentially a diagnosis of exclusion [5].

Non-coronary causes of chest pain include acute life-threatening causes such as pulmonary thromboembolism, acute aortic dissection, tension pneumothorax, and cardiac tamponade, as well as other acute or chronic conditions that are rarely fatal, such as musculoskeletal disorders, digestive tract diseases, psychiatric conditions, neurological disorders [6], rheumatological diseases, or even post-infectious exanthematous diseases [3].

The chest pain caused by ACS is a medical emergency, but the non-cardiac causes of chest pain that are rarely life-threatening can also impact the quality of life [7].

Moreover, the existence of a non-coronary diagnosis that justifies the presence of chest pain can lead to misinterpretation of the chest pain regarding its coronary origin by the patient or medical staff, potentially leading to medical errors that can sometimes be fatal [8]. Some of the pathologies that exhibit chest pain are stated in Table 1. 

Pre-existing documented coronary artery disease or cardiovascular risk factors in patients presenting with non-specific chest pain have a significant impact on their survival, with studies reporting a death rate ranging from 1.4% to 8.1% [9]. One of the possible causes of chest pain is esophageal leiomyoma (EL), a benign tumor of the esophageal musculature. Esophageal leiomyoma is a rare condition of the esophagus. It appears more frequently in men than in women (the incidence ratio is 2:1 in favor of men) [10]. Most frequently it is diagnosed between 20 and 50 years of age [10].

## 2. Case Presentation

We present the case of a 48-year-old female patient, evaluated in the emergency department of our hospital in January 2020 for the first time. She complained of having anterior chest pain with a “claw”-like character radiating to the IVth and the Vth finger of the left hand. Furthermore, she specified that the pain appeared at rest for a couple of minutes and disappeared spontaneously. It also appeared at moderate intensity effort and disappeared with cessation of effort. She mentioned that dyspnea, tachycardia, and irregular palpitations accompanied the thoracic pain. The painful episodes were preceded by profuse sweating and followed within a few minutes by headache and nausea. The patient also stated that she had daytime fatigue, marked anxiety, fear of impending death, sleep-onset insomnia, and huge blood pressure variation upon home blood pressure monitoring.

Personal history: We note the following physiological antecedents: menarche at 15 years, menopause at 45 years, having 3 children from 3 pregnancies each with a birth weight under 4000 g, and the subsequent personal pathological history: essential hypertension (onset at the age of 38, with the highest recorded blood pressure value being 195/105 mmHg). The patient had the following CV risk factors: arterial hypertension, young age of onset of arterial hypertension in a female patient, first-degree obesity (BMI = 32 kg/m^2^ sc), dyslipidemia, and elevated homocysteine values without being a smoker (the patient was a lifetime non-smoker) and non-drinker, with no significant heredity for cardiovascular events.

She worked in a factory in alternating shifts (8 h each) without exposure to occupational hazards. This professional activity involved flexion and extension of the cervical spine and repetitive movements of the fingers and hand (assembling cables for the automotive industry).

The clinical examination was without any pathological finding, and the patient was temporo-spatially oriented. Other factors included blood pressure (BP) = 150/90 mmHg (right arm) and 140/100 mmHg (left arm), heart rate (HR) = 80 bpm, regular pulse, weight = 77 kg, height = 153 cm, and abdominal circumference = 105 cm.

**Figure 1:**
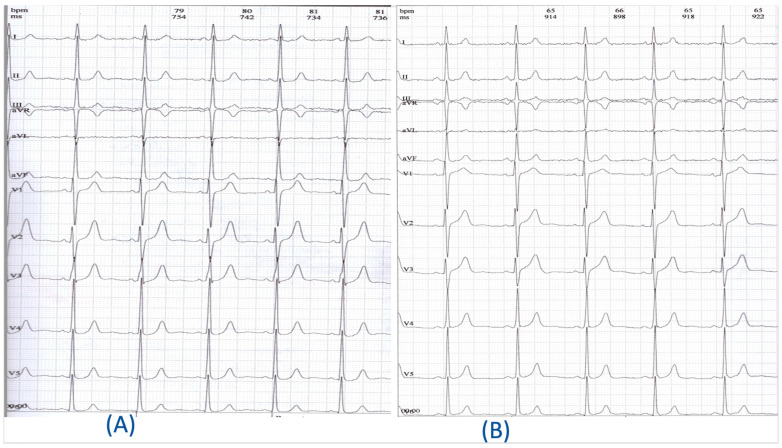
(**A**) Resting ECG: the patient’s ECG was recorded at the moment that the patient complained of chest pain (25 mm/s, 10 mm/1 mV). (**B**) ECG performed one year after the surgical intervention (ECG = electrocardiogram).

Resting electrocardiography (ECG) performed during her first visit to our Outpatient Clinic showed the presence of regular sinus rhythm without any changes suggestive of myocardial ischemia (Figure 1A).

Transthoracic echocardiography documented a normal systolic function of the left ventricle (LV), type I diastolic dysfunction of LV, normal cavity and normal wall dimensions, degenerative valvular changes in the mitral and aortic valves, mild degenerative mitral regurgitation, and no segmental or global wall motion abnormalities.

**Figure 2:**
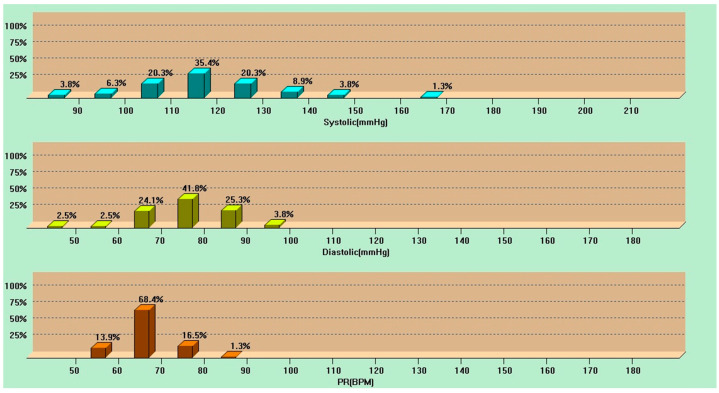
Ambulatory blood pressure monitoring over 24 h (X-axis shows blood pressure values and the Y-axis shows the percentage of time within the X-axis blood pressure value (upper part systolic blood pressure and middle part diastolic blood pressure). X-axis shows pulse rate values in beats per minute (BPM) and the Y-axis shows the percentage of time within the X-axis pulse rate (lower)).

Ambulatory blood pressure monitoring over 24 h indicated unsatisfactory control of blood pressure values (Figure 2).

Treatment was initiated with bisoprolol 2.5 mg/day, isosorbide mononitrate 15 mg on the first day increasing by 15 mg/day to reach the target dose of 60 mg/day, clopidogrel 75 mg/day, zofenopril 2 × 7.5 mg/day, doubling the dose every 7 days until reaching a target dose of 2 × 30 mg/day, acetylsalicylic acid 75 mg/day, and atorvastatin 40 mg/day.

Laboratory evaluations reveal the following altered parameters: erythrocyte sedimentation rate (ESR) = 15 mm/h, fasting blood glucose (FBS) = 120.98 mg/dL, total cholesterol = 228.27 mg/dL, HDL cholesterol = 63 mg/dL, LDL cholesterol = 148.4 mg/dL, TSH = 4.063 mUI/L (normal range 0.4–3.6 MUI/L), and homocysteine = 15.3 µmol/L.

The following parameters were within normal limits: total serum triglycerides = 121 mg/dL, complete blood count, serum iron, AST, ALT, FT3, FT4, serum fibrinogen, serum potassium, serum sodium, total serum bilirubin, serum creatinine, serum urea, alkaline phosphatase, gamma-glutamyl transferase, total CK, CK-MB, troponin I, high-sensitive CRP, and NT-pro-BNP.

**Figure 3:**
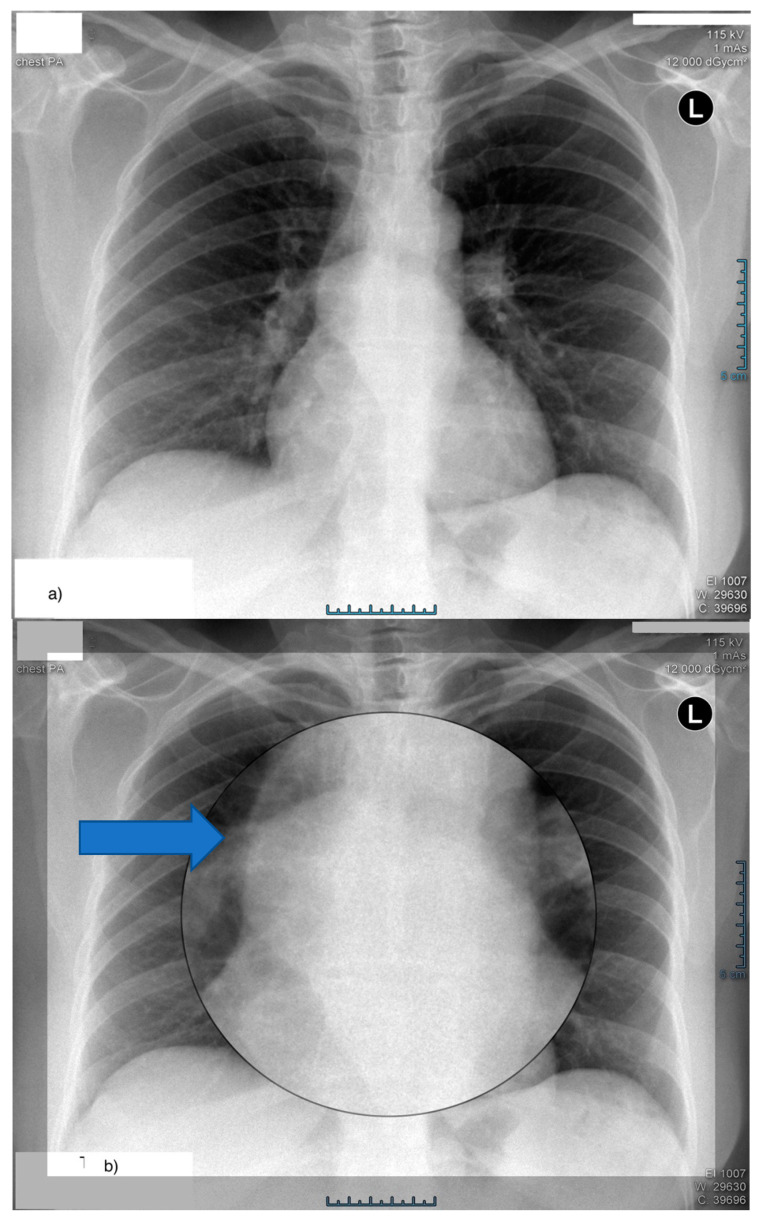
Posterior–anterior chest X-ray: (**a**) non-homogeneous mediastinum with the highlighting of an oval structure of super-costal intensity widening the tracheal bifurcation angle, (**b**) enlarged image of the mentioned structure. The blue arrows point to the enlarged structure.

Chest X-ray (Figure 3a,b) showed the image of a non-homogeneous mediastinum with the widening of the tracheal bifurcation angle, and spirometry (Table 2) was normal.

The patient refused to remain admitted to the hospital and left the emergency unit, on discharge against medical advice (DAMA), with this being the only presentation in the Emergency Department until the time of surgery. At her presentation to the Emergency Department, myocardial enzymes and ECG were within normal limits, but the pain had a clear description of anginal pain, thus being classified as atypical angina, according to the ESC classification [11], following the first presentation in the Cardiology and Internal Medicine Clinic. Dual antiplatelet administration was chosen, considering the patient to be at high risk of ischemic events (Class IIa, Level B indication—recommendations for event prevention I, according to [11]), to be reconsidered after obtaining the data of the additional investigations recommended at the first presentation of the patient in our service (Cardiology and Internal Medicine Outpatient Clinic). Thus, at the first outpatient presentation, the patient was classified as having stable angina pectoris, and biological and paraclinical evaluation was initiated and treatment was instituted.

**Figure 4:**
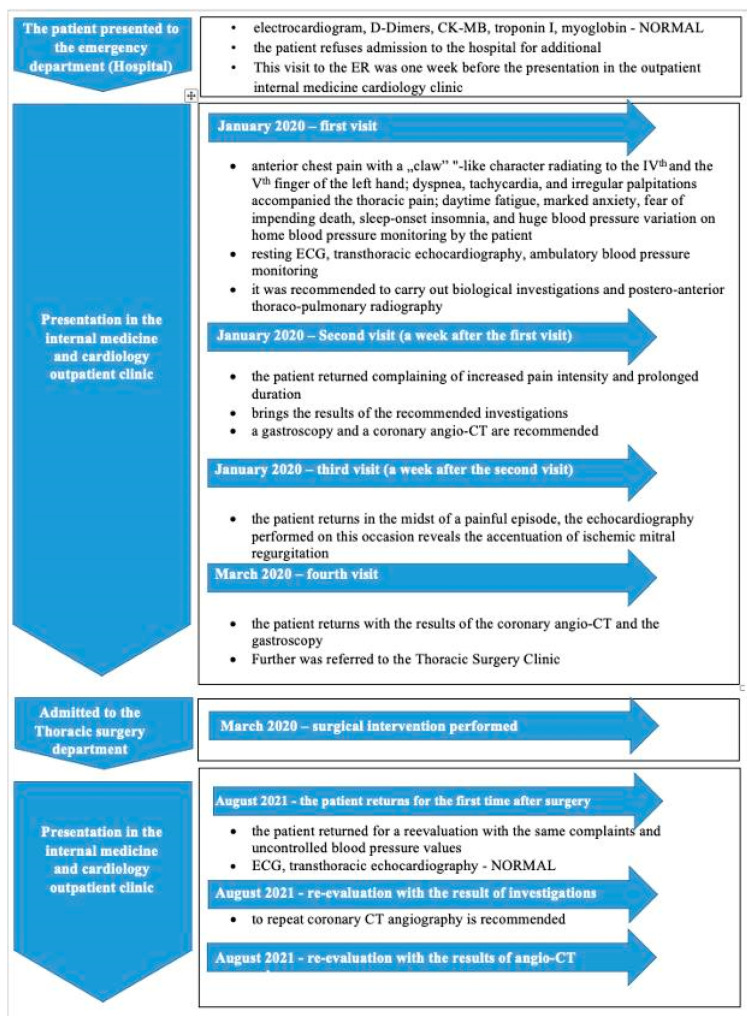
Timeline of events.

A week after the first evaluation in our Outpatient facility, the patient returned to our internal medicine and cardiology Outpatient Clinic complaining of increased pain intensity and prolonged duration. ECG and transthoracic echocardiography were repeated, showing no changes from the previous presentation. Trazodone hydrochloride 75 mg was added in the evening to the previously established treatment plan. The timeline of the events is presented in Figure 4.

Furthermore, one week later, the patient was evaluated in our Outpatient Clinic during a full episode of chest pain, revealing increased mitral regurgitation and its eccentric orientation.

At the second presentation, the patient showed the result of the recommended investigations, objecting to the mediastinal mass on the chest X-ray at this moment but describing the increase in the intensity of the pain, as well as its prolongation. These two elements (chest X-ray and the change in the clinical characteristics of the chest pain) were the basis of the decision to send the patient to undergo a coronary angio-CT (to see both the actual state of the coronary arteries and the structures adjacent to the heart) and gastroscopy to highlight the esophagus. The patient’s return to the Outpatient Clinic after another week with full chest pain and the ischemic-type change in the echocardiographic appearance of the mitral regurgitation made us suspicious of angina pectoris.

**Figure 5:**
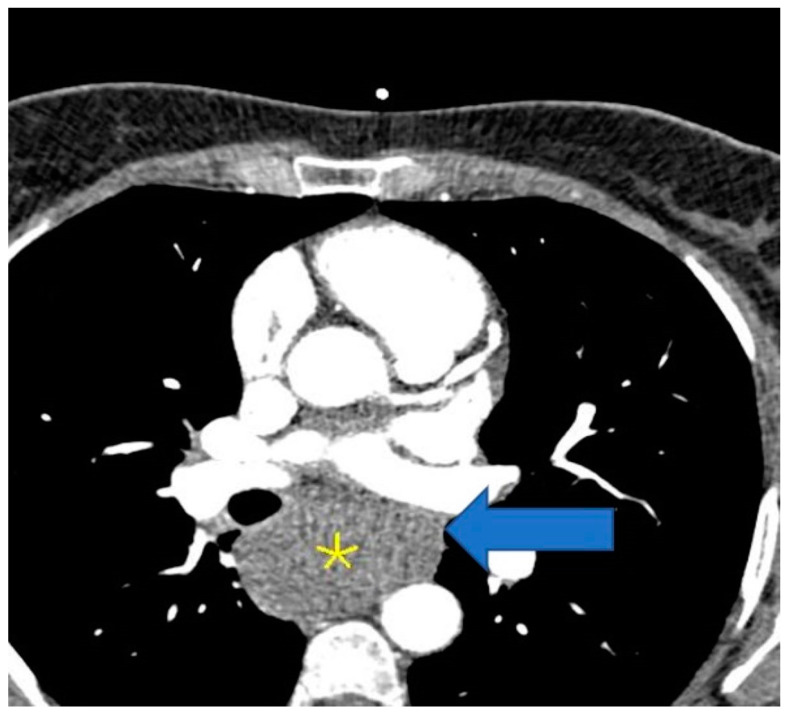
CT thorax with contrast (for coronary angiography) showing a solid mass enveloping the esophagus (yellow star).

**Figure 6:**
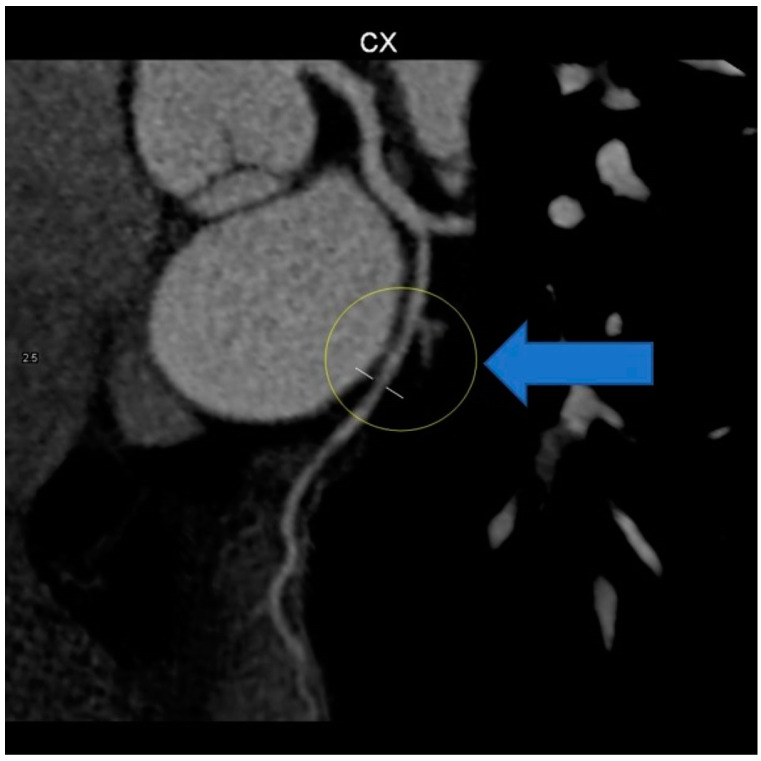
CT coronary angiography showing normal appearance at the circumflex artery level (yellow circle).

The patient was referred to the radiology department for computed tomography (CT) angio-coronarography, which revealed a voluminous mass situated into the mediastinum oriented towards and beneath, encompassing the esophagus and compressing it. The described mass measured 57 mm in diameter and was in direct contact with the carinal. Internal densities of 71 HU ruled out the presence of lipoma or mediastinal cyst, suggesting an esophageal mass or (less likely) fused lymph node block. The total Ca score was 7.7 UA and there was a single calcific spot in the middle segment of the right coronary artery (RCA), with no significant predictive risk for acute coronary events. The heart was predominantly supplied by the RCA, with the normal origin and initial course of the coronary arteries. There was dominant RCA, patent, with a single 2 mm calcific spot in the middle segment, no significant stenosis, and patent otherwise. The left main (LM) is bifurcated and patent. Left anterior descending (LAD) and diagonals were without atheromatosis, with no stenosis or occlusions. The left circumflex coronary artery (LCX) was patent. The aortic valve was tricuspid aortic, with normal function. Internal mammary arteries, pulmonary arteries, and veins were normal, without thrombosis or abnormal drainage. Ascending aorta had normal diameter and flow, without any pathological changes in the left ventricular myocardium or the left cardiac chambers on computed tomography (Figure 5 and Figure 6).

Given the result of the CT coronary angiography, an upper endoscopic evaluation was performed, revealing an esophagus with a normal mucosal appearance, showing an aspect of extrinsic compression starting at about 25 cm from the dental arch, a stomach without changes, congested antral mucosa, and normal duodenal bulb and DII portion. Almost 3 months after the first presentation in the Outpatient Clinic, we outlined the diagnosis of “Esophageal parietal tumor mass” for which the patient was referred to the Thoracic Surgery service.

Considering these elements, in March 2020, the patient was referred to the Thoracic Surgery department with the diagnosis of a mediastinal tumor mass enveloping the esophagus. The patient was admitted to the thoracic surgery department where a left postero-lateral mini-thoracotomy in the sixth intercostal space was performed, identifying a well-defined esophageal tumoral mass resecting the mucosal and submucosal layers, without signs of local invasion; complete enucleation of the tumoral formation was performed through extended esophagostomy and sent for morpho-pathological examination. The morpho-pathological examination indicated the presence of esophageal fibro leiomyoma.

After surgery, the patient did not return to our service for further evaluations, although she was medically advised to come for further follow-up. She also discontinued antihypertensive, antiplatelet, and statin therapy on her own initiative, maintaining trazodone hydrochloride for treating sleep-onset insomnia.

In August 2021, the patient returned for a re-evaluation with the same complaints and uncontrolled blood pressure values to our Outpatient Clinic.

The ECG performed on this occasion showed no changes compared to the ones performed a year ago, appearing normal (Figure 1B).

Transthoracic echocardiography was without changes compared to the previous examination. Biological re-evaluation was recommended, and an attempt was made to reassure the patient, who, upon hearing that the cardiac evaluation showed no changes compared to the previous year, was afraid about the recurrence of the esophageal tumor. The previously recommended therapeutic regimen was reintroduced.

During the re-evaluation with the results of the biological investigations, the patient showed abnormal values for homocysteine (13.42 µmol/L, normal value <10 µmol/L), NT-pro-BNP (165 pg/mL, normal value <125 pg/mL), 25-hydroxy-vitamin D (21.8 µg/L, normal value >30 µg/L), LDL cholesterol (142.7 mg/dL), and TSH (3.822 mUI/L, normal value 0.4–3.6 mUI/L). The HDL cholesterol value was 77.2 mg/dL. Values for high-sensitive CRP, aldosterone, plasma adrenaline, plasma noradrenaline, plasma dopamine, serum cortisol, free triiodothyronine, apolipoprotein A1, apolipoprotein B, VLDL cholesterol, lipoprotein (a), serum fibrinogen, complete blood count, serum uric acid, and FT4 were within normal limits.

**Figure 7:**
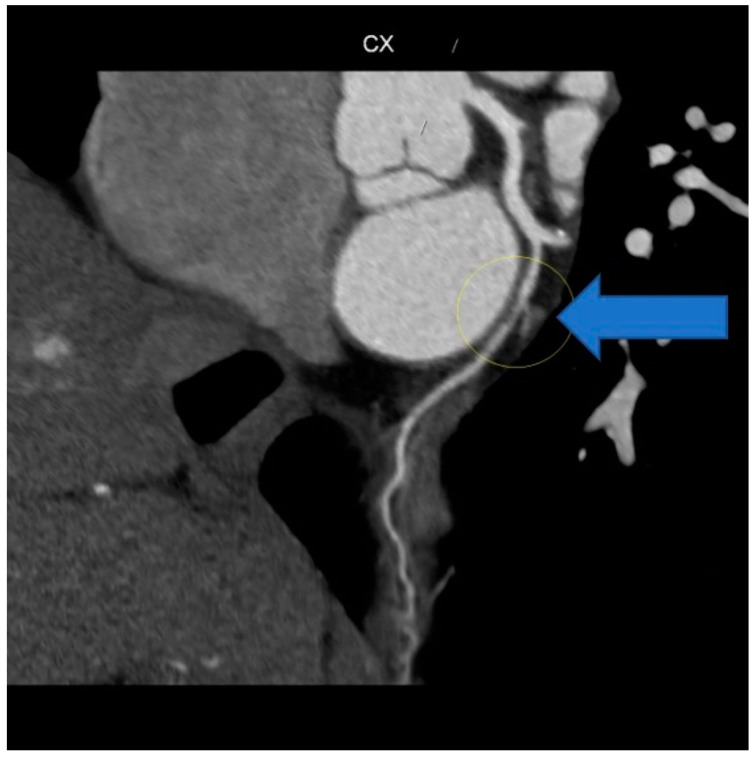
CT coronary angiography showing stenosis (arrow).

Due to the marked anxiety of the patient and the characteristics of the chest pain, a new multi-slice 128 × 0.5 mm ECG-modulated CT coronary angiography was planned, which revealed an average heart rate of 57 bpm; normal coronary artery origin; right-dominant coronary system; dominant RCA with a single calcific spot in the proximal segment, no significant stenosis; posterior descendent artery (PDA), posterior left ventricular artery (PL), and ramus intermediaris (RM) without stenosis; no atheromatous plaque; short, bifurcated LM, without stenosis; LAD and diagonals patent, without stenosis, no atheromatous formation; CX with a stenotic lesion in the middle segment, downstream of obtuse marginal artery (OM) origin, due to fibro-lipid plaque, approximately 50–60%; and OM with mixed stenotic atheromatous plaque at origin, 20–25%, while the rest of the course was patent, without stenosis (Figure 7).

Given the aspects revealed by CT coronary angiography, percutaneous coronary angiography was indicated, which the patient refused, requesting medical treatment instead. The therapeutic regimen was adjusted as follows: bisoprolol 2.5 mg/day, candesartan 8 mg/day, amlodipine 5 mg/day, clopidogrel 75 mg/day, atorvastatin 80 mg/day, acetylsalicylic acid 75 mg/day, and nitroglycerin as and when needed (SOS).

Currently, the patient is stable in terms of coronary symptomatology, with the effort threshold at which angina pectoris appears being classified in the Canadian class II of stable angina pectoris.

## 3. Discussion

This case presents a patient who refused hospital admission and who was evaluated in a cardiology Outpatient Clinic for chest pain with multiple characteristics that could frame it as of coronary origin. Initially, the presence of coronary artery disease was excluded by performing a coronary angio-CT, which indicated the presence of an esophageal tumor mass (incidental finding). After the diagnosis of esophageal leiomyoma and its surgical treatment, the patient was asymptomatic. Approximately one year after the surgical intervention, following the cessation of antiplatelet therapy and statin, the patient returned for the evaluation of reappearing chest pain with the same characteristics as previously presented, terrified by the possibility of the recurrence of the esophageal leiomyoma. Upon repeating investigations, it was proven through coronary angio-CT evaluation that, this time, the origin of the chest pain was indeed coronary. However, the patient still refused hospital admission and the performance of percutaneous coronary angiography.

Esophageal leiomyoma in the majority of cases is an incidental finding, although it is one of the most common forms of esophageal tumors, with a higher incidence in men (with the male-to-female ratio being 2:1). They are predominantly located in the lower third of the esophagus, followed by the middle third, and very rarely in the upper third (up to 7% of cases described in the literature) [10,12]. Our case presented with the placement of EL in the upper third with an extension into the middle third of the esophagus.

The most frequently encountered symptoms in patients with large tumors (whose incidence is also rarer) are digestive type (epigastric pain, dysphagia, regurgitation, or gastrointestinal bleeding and, more rarely, diarrhea or weight loss) [13,14] and chest pain, which is related to the esophagus (the situation in which it is located laterally to the right edge of the sternum). In our case, the main manifestation was chest pain with coronary characteristics (occurring at moderate-intensity efforts and disappearing after stopping the effort, located retrosternally) without epigastric radiation and associated with dyspnea and rarely nausea. A higher incidence of EL is reported in smokers or patients with hiatal hernia [14], but none of these situations were present in our patient and she was a non-smoker. Additionally, in women, an association with other locations is frequent, and the uterine site is frequently described [14], but gynecological evaluation of our patient did not reveal any pathological changes. Intimate contact with the tracheal carina can explain the presence of dyspnea, rarely reported among the symptoms present in patients with EL and only in patients with giant tumor formations [15], in addition to the lack of changes at the spirometry level.

Even if the imaging of choice for cases with a high suspicion of the presence of an esophageal tumor is barium swallow-guided chest radiography followed by computed tomography, without excluding the possibility of endoscopic evaluation of the upper digestive tract [16], we decided on endoscopic evaluation of the upper digestive tract and the performance of CT coronary angiography. The decision was based on the previously performed radiological evaluations and the likeliness of the symptomatology being cardiovascular in origin. Obtaining an accurate diagnosis and excluding coronary etiology from the first presentation was our prime target.

With the medical data obtained during the first evaluation, the recurrence of chest pain could be investigated again for coronary etiology using CT coronary angiography, and by comparing the two results, we could exclude local recurrence of EL and highlight the appearance of coronary stenosis that could be responsible for the chest pain presented at this time.

The discontinuation of statin and antiplatelet therapy enacted by the patient without medical approval might have aggravated the blockage and the appearance of coronary stenosis on two important vessels, LCX and OM. If the patient had continued the treatment, this situation could have possibly been avoided.

Medical data regarding the link between statin cessation and the occurrence of acute coronary syndromes or worsening of chronic coronary syndromes and increased mortality are clear at this moment, leading to discussions about statin withdrawal syndrome [17,18,19,20,21].

The discontinuation of statin therapy is associated with inducing or exacerbating vascular dysfunction, the rebound of inflammatory processes suppressed by the use of “potent” statins, and reducing the process of angiogenesis, which directly affects the myocardium and may be associated with a decrease in the angina threshold or worsening of coronary pain characteristics [19,21].

The statin treatment regimen, even for patients with non-obstructive coronary artery disease (stenosis ≤ 50%), has been shown to reduce the risk of myocardial infarction and all-cause mortality, being directly dependent on coronary artery disease burden [22].

On the other hand, the discontinuation of low-dose aspirin therapy in patients with a history of cardiovascular disease or with present cardiovascular risk factors is associated with a higher risk of worsening angina or non-fatal myocardial infarction than those who continued treatment, as well as the risk of other thrombotic events [23,24]. The risk of thrombotic phenomenon after the discontinuation of acetylsalicylic acid therapy occurs without a protective period from its cessation and increases from one day to the next [25] even though platelet aggregation capacity recovers almost entirely in 3–4 days [26].

The increased risk of thrombotic phenomena after the discontinuation of aspirin therapy seems to be due to elevated thromboxane A2 levels [25] and a rebound phenomenon of platelet aggregation [26]. Aspirin is considered one of the key elements in the secondary prevention of cardiovascular events, while it should be considered an important drug in primary prevention due to its properties. Because physicians often suffer the dilemma of initiating these drugs or not due to their atherothrombosis vs. bleeding risk, proper screening protocols and risk stratification guidelines can help encourage doctors to use these drugs more prudently [27].

The combination of aspirin–statin therapy is significantly superior to aspirin administration alone for the prevention of cardiovascular diseases [28], and the lack of adherence to these treatments is associated with premature atherosclerotic cardiovascular disease [29] and increased mortality [30].

Chest pain of any origin cannot be left unattended as underlying pathologies can be fatal. In a meta-analysis conducted on different causes of chest pain and patients’ presentation to the primary care units, it was seen that the majority had coronary heart disease [31]. The timely initiation of therapy can not only help save the lives of the patients but also improve the quality of life.

One of the major shortcomings of the Outpatient approach to this patient is the long time that elapsed between the presentations and the performance of the recommended investigations, as her admission to the hospital would certainly have shortened the period of time until the correct treatment was carried out. An aspect worth mentioning is that the investigation of the patient and the surgical intervention took place in the midst of the epidemiological uncertainty of the evolution of the COVID-19 pandemic. Hence, the exercise test evaluation was not performed, nor was the 12-channel/24 h Holter EKG monitoring. Both of these investigations could have saved time and helped in obtaining significantly important medical information, as well as performing coronary angiography.

The implemented restrictions during the lockdown led to further delays in the establishment of the diagnosis. Furthermore, the financial aspect of the investigations is worth mentioning because the Romanian National Health Insurance does not cover the costs of coronary angio-CT.

## 4. Conclusions

Chest pain is a symptom that appears in multiple pathologies, from those without an effect on patient survival culminating in mortality from myocardial infarction. Any chest pain should be meticulously approached, and among the first diagnoses to be excluded are life-threatening ones such as myocardial infarction and pulmonary thromboembolism. In our case, the patient’s initial presentation with typical coronary chest pain was proven to be of non-coronary origin, and surgical treatment of the incidentally found esophageal tumor resulted in the disappearance of chest pain. The absence of chest pain led the patient to stop cardiovascular treatment (which was prescribed due to the typical characteristics of the pain based on guidelines), which might have further aggravated the blockage of the vessels leading to the reappearance of chest pain, which, this time, was proven to be of coronary origin. The importance of continuing treatments proven to reduce cardiovascular mortality should be emphasized by physicians, regardless of their specialty, and the indication for continuation should be mentioned on the medical documents received by the patient. Any case can hold a unique out-of-the-box presentation, hence thorough investigations leaving no stone unturned should be the approach of a good doctor, irrespective of their specialty.

## Figures and Tables

**Table 1:** Differential diagnosis of chest pain based on location.

Origin of Pain	Pathologies	Investigations
Psychogenic	Panic disorder Anxiety Somatization Cocaine abuse	History Psychological and psychiatric evaluation Cocaine confirmatory test
Neurogenic	Herpes zoster Neuropathies Radiculopathy at the thoracic level Thoracic outlet syndrome Disc prolapse	Neurological evaluation X-ray CT-scans MRI PCR for Herpes zoster
Pulmonary	Pneumothorax Pleuritic pain Pulmonary emboli Pneumonia Pulmonary hypertension Tuberculosis Asthma Malignancy Bronchospasm	X-ray Lung ultrasound CT-scan MRI Spirometry Laboratory investigations Mountoux skin test Sputum analysis
Cardiovascular	Takotsubo cardiomyopathy Cardiac tamponade Pericarditis Myocarditis Aortic stenosis Mitral valve prolapse Acute coronary syndrome Stable angina Aortic aneurysm Aortic dissection	X-ray Echocardiography ECG Angio-CT Angiography Laboratory investigations
Gastrointestinal	Esophagitis Esophageal spasm Achalasia GERD Cholangitis Cholecystitis Peptic ulcer Pancreatitis Tumors	Barium swallow Endoscopy Abdominal ultrasound Laboratory investigation
Musculoskeletal	Cervical pathologies Rib fracture Costochondritis Arthritis—sternoclavicular Rheumatic disease	X-ray CT-scans MRI

**Table 2:** Main parameters of the spirometry results of the patient, performed at the time of first presentation—normal aspect of the flow/volume curves.

Parameter	LLN	ULN	PRE #1	PRE #1%	PRE %	Z-Score	POST #1
FVC	1.93	3.35	2.64	2.84	108	0.47	2.78
FEV1	1.62	2.87	2.24	2.43	108	0.49	2.4
FEV1/FVC	69.3	90.7	80	85.6	107	0.86	85.3

## Data Availability

Data will be made available for valid written requests addressed to the corresponding author.
